# Genetic and brain similarity independently predict childhood anthropometrics and neighborhood socioeconomic conditions

**DOI:** 10.1016/j.dcn.2023.101339

**Published:** 2024-01-04

**Authors:** Andreas Dahl, Espen M. Eilertsen, Sara F. Rodriguez-Cabello, Linn B. Norbom, Anneli D. Tandberg, Esten Leonardsen, Sang Hong Lee, Eivind Ystrom, Christian K. Tamnes, Dag Alnæs, Lars T. Westlye

**Affiliations:** a Department of Psychology, University of Oslo, Oslo, Norway; b NORMENT, Division of Mental Health and Addiction, Oslo University Hospital & Institute of Clinical Medicine, University of Oslo, Oslo, Norway; c Research Center for Developmental Processes and Gradients in Mental Health (PROMENTA), Department of Psychology, University of Oslo, Oslo, Norway; d Department of Psychiatric Research, Diakonhjemmet Hospital, Oslo, Norway; e Australian Centre for Precision Health, UniSA Allied Health & Human Performance, University of South Australia, Adelaide, Australia; f South Australian Health and Medical Research Institute (SAHMRI), University of South Australia, Adelaide, Australia; g Department of Mental Disorders, Norwegian Institute of Public Health, Oslo, Norway; h KG Jebsen Center for Neurodevelopmental Disorders, University of Oslo, Norway

**Keywords:** Brain similarity, Morphometricity, Cortical Thickness, ABCD study, SNP heritability

## Abstract

Linking the developing brain with individual differences in clinical and demographic traits is challenging due to the substantial interindividual heterogeneity of brain anatomy and organization. Here we employ an integrative approach that parses individual differences in both cortical thickness and common genetic variants, and assess their effects on a wide set of childhood traits. The approach uses a linear mixed model framework to obtain the unique effects of each type of similarity, as well as their covariance. We employ this approach in a sample of 7760 unrelated children in the ABCD cohort baseline sample (mean age 9.9, 46.8% female). In general, associations between cortical thickness similarity and traits were limited to anthropometrics such as height, weight, and birth weight, as well as a marker of neighborhood socioeconomic conditions. Common genetic variants explained significant proportions of variance across nearly all included outcomes, although estimates were somewhat lower than previous reports. No significant covariance of the effects of genetic and cortical thickness similarity was found. The present findings highlight the connection between anthropometrics as well as neighborhood socioeconomic conditions and the developing brain, which appear to be independent from individual differences in common genetic variants in this population-based sample.

## 1 Introduction

Mapping individual differences in brain morphology and their associations with relevant clinical and demographic traits has been described as one of the fundamental challenges of neuroscience (Giedd and Rapoport, 2010, Lashley, 1947). This task is particularly challenging in young individuals, as the structure of the brain changes rapidly when they progress through different stages of development (Mills et al., 2021). A morphological measure that has received extensive attention is the thickness of the cortex, both due to its potential sensitivity to age (Frangou et al., 2022) and clinical conditions (Hettwer et al., 2022). Cortical thickness can be estimated from magnetic resonance imaging (MRI) data with reasonable accuracy (Dale et al., 1999). However, reported associations between apparent cortical thickness and observable traits in children and adolescents often differ between studies (Ferschmann et al., 2022). This lack of robustness may in part be attributed to a methodological reliance on average effects that inadequately accounts for the individual heterogeneity of cortical structure and development, as well as a lack of consideration of interactions between brain development and individual differences in genetic and social makeup (Ferschmann et al., 2022). This has instigated a call for new approaches that better capture individual-level variability in brain development and its links to genetic and environmental influences (Bottenhorn et al., 2023, Foulkes and Blakemore, 2018).

In the field of genetics, leveraging the inherent genetic similarity among individuals to explore the relationship between their genetic make-up and observable traits has revealed novel insight into the associations between genetic factors and human traits (Tam et al., 2019). For this, a genomic relatedness matrix (GRM) can be constructed (J. Yu et al., 2006) by estimating the pairwise resemblance of individuals in a sample based on genome-wide single nucleotide polymorphisms (SNPs; Yang et al., 2010). This GRM can then be integrated into linear mixed models (LMMs) along with phenotypic traits, enabling the estimation of the proportion of phenotypic variance attributed to genetics (commonly known as SNP-based heritability). This approach, often referred to as genome-based restricted maximum likelihood (GREML), has been successfully applied to large cohorts of young individuals to estimate SNP-based heritability for complex behavioral traits, such as academic performance, psychological distress, and externalizing behavior (Cheesman et al., 2020, Donati et al., 2021, Eilertsen et al., 2022, Jami et al., 2022). The application of GREML in neuroscience has been limited compared to other fields (Trzaskowski et al., 2014), and between-subject variability is commonly considered as error. There is a growing recognition of the potential benefits of incorporating individual variability caused by genetics to enhance our understanding of the relationship between the developing cortex and observable traits (Z. Yu et al., 2022).

Applying a similar approach to GREML, Sabuncu et al. (2016) reported that a significant proportion of the variance of both clinical (e.g., diagnosis of mental illness) and non-clinical traits (e.g., cognition) could be explained by whole-brain morphology, specifically a composite of multiple gray- and white matter measures in adults. This approach has been termed *morphometricity* (Sabuncu et al., 2016), or *trait morphometricity* (Fürtjes et al. (2023). In simplified terms, this approach entails estimating one or more measures of brain morphology, such as cortical thickness and cortical surface area. Next, the pairwise resemblance across all vertices or regions of interest (ROIs) of one or more such morphological measures are calculated across all individuals, resulting in a brain-morphological similarity matrix, analogous to a GRM. The resulting matrix is then used in LMMs, yielding an estimate of the proportion of phenotypic variance attributed to brain morphology. This approach has been expanded by Couvy-Duchesne et al. (2020) and Fürtjes et al. (2023), showing that similarity matrices based on morphological measures explain significant proportions of variance across different groups of traits, such as anthropometrics (e.g. BMI), cognition, markers of socioeconomic status and health behaviors (Couvy-Duchesne et al., 2020). Importantly, comparisons have shown that similarity-based approaches consistently outperformed conventional univariate association analyses, both in terms of power to detect effects and in explained trait variability, in clinical and non-clinical traits (Sabuncu et al., 2016).

The current study expands on this work by investigating both the morphometricity and SNP-based heritability of a wide array of traits in a large sample of US children from the Adolescent Brain Cognitive Development (ABCD) study cohort baseline sample. While we are not aware of any previous study investigating the morphometricity of traits in younger individuals, it is conceivable that this approach might manifest differently in children compared to adults. We will restrict our approach to cortical thickness, which shows marked changes during development (Fuhrmann et al., 2022), and is reasonably robust against confounds such as head size and total brain volume (Barnes et al., 2010). By assessing both morphometricity and SNP-based heritability within the same LMM framework, we estimate the observed trait variance that can be explained by both genomic and morphological effects, i.e. a combined genome-morphometric analysis. Our approach also allows for the exploration of the covariance between the genomic and morphological effects, using the CORE GREML approach (Zhou et al., 2020). Traditionally, REML estimation assumes independence between random effects. However, cortical morphology has been shown to be heritable in both adults and younger individuals (Fernandez-Cabello et al., 2022, Shadrin et al., 2021, van der Meer et al., 2020), potentially biasing estimates. By utilizing the CORE GREML approach, we can account for potential dependencies between genomic and morphological random effects, resulting in a more accurate estimation of their respective contributions. In addition, the inclusion of a third term describing the covariance allows for the delineation of the unique contributions of genomic and morphological effects on the trait of interest. This allows us to assess if the potential covariance of their effects manifest differently depending on the trait under investigation.

## 2 Materials and methods

### 2.1 Participants

The full sample for the main analysis following MRI and genetics quality control (QC; see below) consisted of data from 7760 individuals (mean age 9.9 years, 46.8% females) obtained from the ABCD annual data release 3.0 (http://dx.doi.org/10.15154/1523041). The ABCD study (https://abcdstudy.org/) is an ongoing longitudinal developmental study (Volkow et al., 2018) following participants from age ∼10 to age ∼20, with bi-annual collection of brain MRI data. Only data from the baseline session was included in the current analyses. A comparison on key demographic characteristics for participants that did and did not pass QC is given in supplementary table 1. Overall, we noted negligible differences in the sex, age and family income of participants that did and did not pass QC. A small effect size was noted in differences in race / ethnicity. Participants passing QC were somewhat more likely to report their race / ethnicity as white and less likely to report their race / ethnicity as Asian.

### 2.2 Ethical approval

The review and approval of the ABCD research protocol was handled by a central Institutional Review Board at the University of California, San Diego (Auchter et al., 2018). Informed consent was given by parents or guardians and assent was given by children before participation. The present project is registered in the NIMH Data Archive as project number 1467 (doi: 10.15154/1524691), available for registered and authorized users (Request #7474, PI: Westlye). The current project has also been approved by the Norwegian Regional Committee for Medical and Health Research Ethics (REC; #2019/943).

### 2.3 Genetic data - genomic relatedness matrix

Genotyped data was provided by the ABCD consortium, specifically from the Genomics sample_03 (https://nda.nih.gov/study.html?id=1299). A full description of the collection and handling of genotyped data can be found at https://nda.nih.gov/experimentView.html?experimentId= 1194. QC was performed by the ABCD consortium using the RICOPILI pipeline (Lam et al., 2020). Robust relatedness estimates were generated from genotyped SNPs using the *pcrelate* function from GENESIS version 2.24.0 (10.18129/B9.bioc.GENESIS; Conomos et al., 2016), and converted into a GRM using the *pcrelateToMatrix* function from the same package. A GRM describes an estimate of the additive genetic relationship between individuals, where each off-diagonal entry denotes the estimated relatedness for a pair of individuals. It can be expressed as.

$$GRM=\frac{G\times G^\mathsf{T}}{n}$$

Where $GRM$ is the resulting genomic relatedness matrix, $G$ are columns of allele counts standardized to have a mean zero and a standard deviation of one, and $n$ is the number of SNPs. Before the final analysis, for pairs with a familial or cryptic relatedness of 0.05 and above, one individual was removed using the *grm-cutoff* function from GCTA version 1.93.0 (Yang et al., 2011), leaving the maximum possible sample size of non-related individuals.

### 2.4 MRI QC and processing

A full description of ABCD MRI collection and acquisition parameters is given in Casey et al. (2018). Participants that did not pass the recommended image inclusion criteria provided by the ABCD consortium were removed from the sample (imgincl_t1w_include == 0; see http://dx.doi.org/10.15154/1523041 for full details of the QC procedure). T1-weighted MRI data from participants that passed the QC were processed using FreeSurfer 7.1 (surfer.nmr.mgh.harvard.edu). Cortical thickness was computed vertex-wise, as coarser atlas-based ROIs may carry insufficient spatial information for reliable estimates of the morphometricity of traits (Fürtjes et al., 2023). Individual cortical thickness surfaces were registered to a common template (fsaverage) and smoothed using a 15 mm full width at half maximum (FWHM) gaussian kernel. Non-cortical vertices belonging to the medial wall were excluded, leaving a total of 299 879 vertices across both hemispheres for each participant.

To account for scanner-related confounds, a ComBat harmonization procedure was implemented in neuroCombat version 1.0.13 in R (https://github.com/Jfortin1/neuroCombat_Rpackage), using an empirical Bayes location-shift model for all 28 scanners (see supplementary figure 1). All outcome measures were added as covariates for the harmonization procedure to preserve the presumed biological variability of trait outcomes, in addition to sex, age and race / ethnicity. The resulting harmonized cortical thickness measures are a linear combination of the covariates and a scanner-specific residuals modulated by both additive and multiplicative scaling factors (Fortin et al., 2018).

### 2.5 Brain similarity

To determine morphological similarity based on cortical thickness, we calculated the cross-product of the transpose of a matrix containing all vertices of all participants. The formula is equivalent to the calculation of GRM, i.e.

$$BRM=\frac{B\times B^{⊤}}{n}$$

Where *BRM* is the resulting *brain relatedness matrix* (BRM), with each off-diagonal element describing the degree of similarity in morphology between two individuals, *B* is a matrix containing centered and scaled measures of cortical thickness for all vertices, standardized to have mean zero and standard deviation of one and n is the total number of voxels.

### 2.6 Covariance between the effects of genomic relatedness and brain relatedness

To investigate the covariance of the effects of brain measures and genomic data, we used the CORE GREML approach developed by Zhou et al. (2020). CORE GREML extends the concept of genome-based restricted maximum likelihood (GREML) by enabling the estimation of the covariance between two random effects through the product of the Cholesky decomposition of the two relatedness matrices. The purpose of using the Cholesky decomposition is both to allow computational efficacy and to obtain an unbiased estimation of the covariance of the random effects, in this case the effects of genomic and brain relatedness. A detailed description and implementation of the full procedure can be found in Zhou et al. (2020). Briefly, the GRM and BRM matrices were transformed to be positive-definite and subjected to Cholesky decomposition. Subsequently, the product matrix of the Cholesky decompositions of the GRM and BRM was calculated. All the necessary steps of this procedure were implemented in MTG2 version 2.22 (Lee and van der Werf, 2016). Estimates of model parameters for the covariance were obtained by fitting the product matrix, along with the GRM and the BRM, in an LMM (see Model 2 below).

### 2.7 Outcome measures

All outcome measures were taken from ABCD data release 3.0. and handled in R version 4.0.0 (https://cran.r-project.org). We included outcome measures from four different domains: anthropometric, parental / residential, cognitive, and clinical (e.g. potential early markers of mental illness). Detailed descriptions of included instruments are given in Table 1. Pearson correlations of all included outcomes are given in Fig. 2. Anthropometrics such as height and weight are highly heritable (Momin et al., 2023), and previously shown considerable levels of morphometricity in adults, with cortical morphology accounting for approximately 20% of the variation in body mass index (Fürtjes et al., 2023). However, heritability estimates of anthropometric measures tend to be lower during childhood and adolescence (Jelenkovic et al., 2016). It remains uncertain if estimates of morphometricity would be equally reduced. As a growing body of evidence demonstrates associations between perinatal and early-life factors and later brain structure (Alnæs et al., 2020, Walhovd et al., 2023), we also included weight at birth.

**Table 1:** Included outcome variables. n equals the final number of available data following genetic and MRI QC and outlier removal.

Measure	ABCD element name	n	Range	Mean
**Youth Anthropometrics PhenX** (Hamilton et al., 2011)				
Height	anthroheightcalc	7163	44–67″	55″
Weight	anthroweight1-3 lb*	7745	40 - 148 lb	81 lb
**Developmental History Questionnaire** (Kessler et al., 2009, Merikangas et al., 2009)				
Birth weight	birth_weight_lbs	7053	2 - 12 lb	6.7 lb
Age at pregnancy	devhx_3_p	7606	13 - 52 y/o	29.5 y/o
**Parent Demographics Survey PhenX** (Stover et al., 2010)				
Parent education	parent1-2_edu†	7657	13 y. – 21 y.	17.6 y.
**Residental history derived scores** (Fan et al., 2021)				
Area deprivation index	reshist_addr1_adi_wsum	7458	40.6 – 125.8	97.2
Child opportunity index	reshist_addr1_coi_r_ed_nat	7082	1 - 100	60.7
**NIH Toolbox** (Gershon et al., 2013)				
Picture vocabulary	nihtbx_picvocab_uncorrected	7620	57 - 112	84.9
Flanker task	nihtbx_flanker_uncorrected	7580	67 - 116	94.4
Working memory	nihtbx_list_uncorrected	7615	55 - 136	97.2
Card sorting	nihtbx_cardsort_uncorrected	7592	65 - 120	93.1
Pattern recognition	nihtbx_pattern_uncorrected	7619	36 - 140	88.0
Reading	nihtbx_reading_uncorrected	7758	69 - 113	91.0
Fluid intelligence	nihtbx_fluidcomp_uncorrected	7656	52 - 131	91.8
Crystallized intelligence	nihtbx_cryst_uncorrected	7626	64 - 108	87.0
**Child behavior checklist** (CBCL;Achenbach and Verhulst, 2010)				
Internalizing	cbcl_scr_syn_internal_t	7584	33 – 88	48.8
Externalizing	cbcl_scr_syn_external_t	7732	33 - 84	45.9
**Prodromal Questionnaire - Brief Version** (Loewy et al., 2011)				
Pre-psychosis	pps_y_ss_number	7759	0 - 6	1.4
**Sleep Disturbance Scale for Children** (Bruni et al., 1996)				
Sleep disturbance	sleepdisturb1-26p‡	6750	26 - 126	36.9

* Average of three measurements

† Maximum total years of education of either parent

‡ Sum of 26 item Likert scale items

Measures of cognition and general intelligence were included due to their clinical and functional relevance and links to both cortical development and genetics (Estrada et al., 2019). For the remaining included measures, we attempted to capture the associations between morphology, genetic influences, and the family and local environment. This includes markers of neighborhood socioeconomic conditions, which have previously been associated with brain imaging derived phenotypes in the ABCD sample (Alnæs et al., 2020, Hackman et al., 2021, Norbom et al., 2023, Rakesh et al., 2023). Lastly, we included measures of early signs of mental illness, including externalizing and internalizing symptoms.

**Fig. 1:**
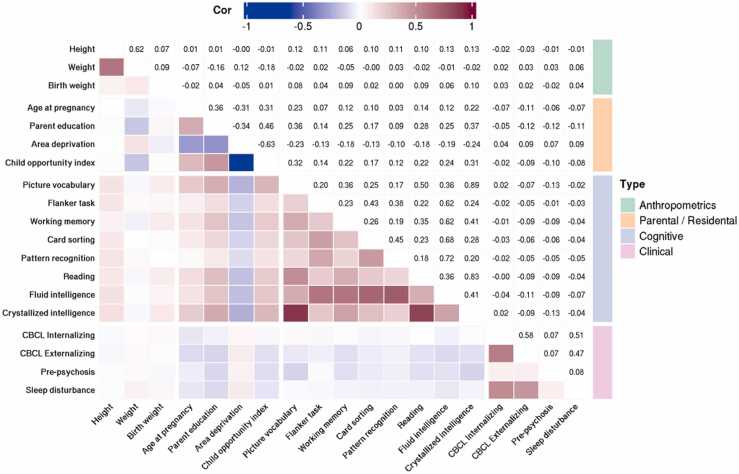
Correlations of all included outcome variables for the final sample (n = 7760). The upper and lower triangular represent the same values, numerically (upper) and color coded (lower).

Outcome scores more than four median absolute deviations from the median were set to missing (Leys et al., 2013). Following this, histograms of outcome distributions were inspected manually, resulting in four weight measurements, all below 40lbs/18 kg., being set to missing. For each outcome variable missing data was removed before being ordered-quantile-normalized using the *bestNormalize* package version 1.8.2. in R (https://cran.r-project.org/package=bestNormalize). Fig. 1.

### 2.8 Data analysis

First, we calculated the overall Pearson’s correlation between the off-diagonal elements of the GRM and the BRM. This correlation provided insights into the similarity or dissimilarity between the two matrices, irrespective of their associations with specific traits. Due to the extensive number of elements, we report descriptive statistics only.

**Fig. 2:**
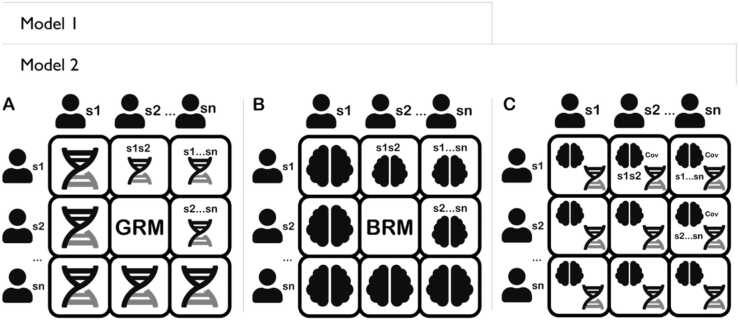
Illustration of random effects included in Model 1 and Model 2. A: Genomic relatedness matrix. B: Brain relatedness matrix. C: Covariance of effects of A and B.

Second, morphometricity and SNP-based heritability estimates were obtained using two separate restricted likelihood random-effects (REML) models for each of the 19 phenotypes (Fig. 2.). The first model is.

$${y} ={X\beta} + {g} + {b} +{\varepsilon}$$

where ${y}$ is the trait of interest, ${X}$ is an incident matrix for the fixed effects ${\beta}$; age, sex, genotype batch, and the first 20 principal components (PCs) of genetic ancestry, to account for population stratification. The 20 PCs were obtained from ABCD Data Release 5.0 (see.

https://data-dict.abcdstudy.org?table_name=gen_y_pihat). ${g}$ is the random genomic effects and $b$ is the random effects of morphological measures. Then, the variance and covariance of $y$ can be written as.

$${var(y)} = {GRM} * {\sigma^2_g} + {BRM} * {\sigma^2_b} + I *{\varepsilon}$$

The second model (i.e. CORE GREML), denoted as Model 2, is the same as in Model 1 except for the addition of the covariance term in the variance and covariance of $y$, which can be written as.

$$var(y) = {GRM} * {\sigma^2_g} + {BRM} * {\sigma^2_b} + I * {\varepsilon} + CORE * cov(g, b)$$

where ${CORE}$ is the product of the Cholesky decomposition of the GRM and the BRM, and $cov(g, b)$ is the covariance between genomic and morphological effects. The GRM and the BRM are identical to Model 1.

The final calculation of morphometricity ($${m^2}$$) is equivalent to conventional heritability ($${h^2}$$) calculation with two random effects, e.g. $m^{2}=\frac{\sigma_{b}^{2}}{\sigma_{b}^{2}+\sigma_{g}^{2}+\sigma_{e}^{2}}$.

is the same as $h^{2}=\frac{\sigma_{g}^{2}}{\sigma_{g}^{2}+\sigma_{b}^{2}+\sigma_{e}^{2}}$.

Estimates of standard error of $m^2$ and $h^2$ were obtained using the Delta method (Oehlert, 1992). If covariance between genomic and morphological effects are present, estimates of ${m^2}$ and ${h^2}$ will be smaller in Model 2 compared to Model 1. Reported p-values are based on Wald tests with one degree of freedom under the null hypothesis that the variance component is zero, implemented in MTG2. Likelihood ratio tests with 1 degree of freedom were performed to determine if the addition of the covariance term significantly improved model fit for a given trait (CORE GREML). The correlation estimates reported in Table 2 is the correlation of the two random effects ${g}$ and ${b}$, obtained as in Zhou et al. (2020) by scaling the covariance by the square root of the product of the variance of the two random effects, i.e.

$$r_{bg} = \frac{{\sigma _{bg}}}{{\sqrt {\sigma _{b}^2 \cdot \sigma _{g}^2}}}$$

**Table 2:** Outcome of log likelihood comparisons of Model 1 and Model 2 and correlations of random effects.

	**Log-likelihood**			**Correlation**		
**Trait**	**log-lkh Model 1**	**log-lkh Model 2**	**p Chi-Square**	**Estimate**	**SE**	**p**
Height	-2718.9	-2718.9	0.937	0.012	0.131	0.941
Weight	-3064.4	-3063.8	0.831	0.171	0.157	0.914
Birth weight	-3010.4	-3009.6	0.831	0.245	0.215	0.914
Age at pregnancy	-3311.9	-3308.9	0.267	0.693	0.356	0.914
Parent education	-2352.1	-2351.0	0.831	0.372	0.268	0.914
Area deprivation	-2760.2	-2759.6	0.831	0.202	0.190	0.914
Child opportunity index	-2164.0	-2163.8	0.937	0.169	0.264	0.941
Picture vocabulary	-2763.6	-2763.3	0.937	-0.205	0.248	0.941
Flanker task	-3554.5	-3554.5	0.937	0.215	0.651	0.941
Working memory	-3300.6	-3300.6	0.937	-0.048	0.274	0.941
Card sorting	-3442.7	-3441.8	0.831	0.567	0.478	0.914
Pattern recognition	-3536.9	-3536.9	0.937	-0.127	0.333	0.941
Reading	-3215.4	-3215.3	0.937	-0.107	0.248	0.941
Fluid intelligence	-3102.2	-3102.2	0.937	0.030	0.207	0.941
Crystallized intelligence	-2755.8	-2755.6	0.937	-0.135	0.203	0.941
CBCL Internalizing	-3617.5	-3617.5	0.937	-0.272	0.917	0.941
CBCL Externalizing	-3564.6	-3564.4	0.937	-0.549	1.019	0.941
Pre-psychosis	-2231.5	-2231.5	0.937	-0.060	0.810	0.941
Sleep disturbance	-3804.5	-3804.4	0.937	N/Aᵃ	N/Aᵃ	N/A^a^

a Did not converge at 20 000 iterations. Estimates unreliable

The sampling variance of the correlation estimates was obtained using the Delta method with variance and covariance terms from Model 2 (CORE GREML).

All reported p-values for variance components, likelihood ratio tests and correlations were adjusted for multiple tests by using false discovery rate (FDR; Benjamini and Hochberg, 1995).

## 3 Results

### 3.1 Gross association of GRM and BRM elements

**Fig. 3:**
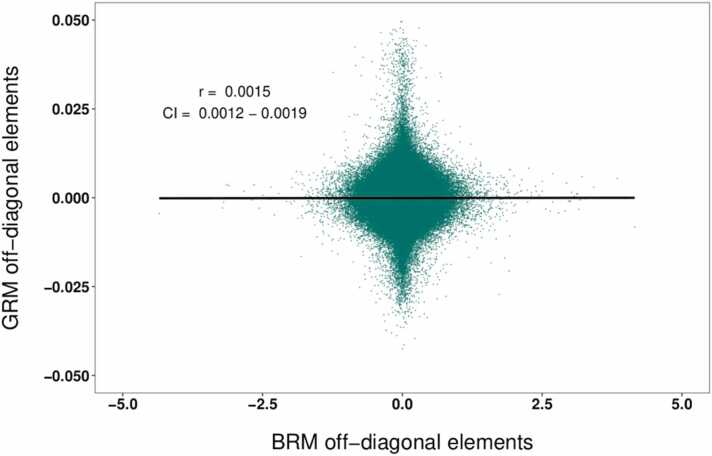
Pearson correlation of the off-diagonal elements of the GRM and the GRM. Scatter shows a random selection of 1 000 000 associations, values indicate the overall r and CI for all 30 104 920 associations.

Correlation analyses revealed a near-zero association between the off-diagonal elements of the GRM and the BRM (r = 0.0015; 95% confidence interval [CI] = 0.0012, 0.0019), indicating that similarity in cortical morphology in children is generally not associated with genomic similarity (Fig. 3).

### 3.2 Model 1

**Fig. 4:**
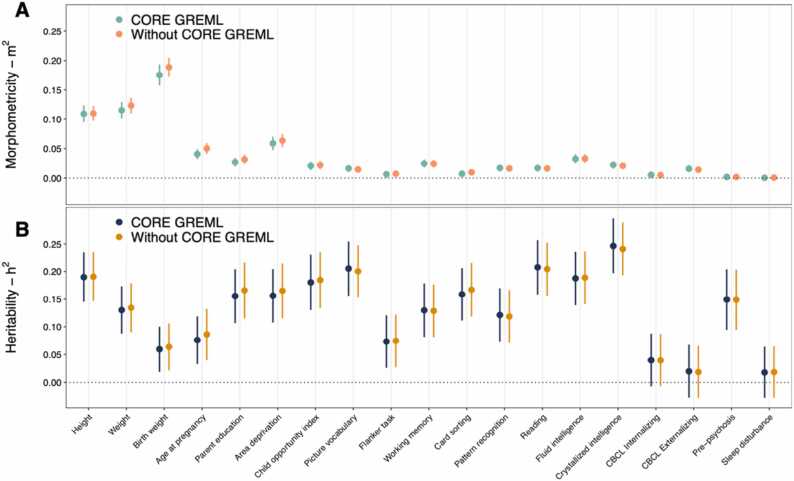
**:** Outcomes of main analysis. (a) $m^2$ estimates and SE for all included traits, either with or without the covariance (CORE GREML)-term included in the LMM. (b) ${h^2}$ estimates and SE for all included traits, either with or without the covariance term included in the LMM.

The full results of Model 1 analyses are presented in Fig. 4 and Supplementary Table S2. The estimates of SNP-based *h*^2^ were significantly different from zero for the majority of included traits. However, it should be noted that the contribution of genetic factors was generally modest and estimated SNP-based *h*^2^ did not exceed 0.30 for any trait. The highest estimates were found for the NIH Toolbox crystallized intelligence composite score (*h*^2^ = 0.24), the NIH Toolbox reading task (*h*^2^ = 0.20), and height (*h*^2^ = 0.19). Genomic similarity was not significantly associated with birth weight, mother's age at pregnancy, the NIH Toolbox flanker task, internalizing and externalizing symptoms or sleep disturbance (all *h*^2^ < 0.1).

The morphometricity analyses revealed associations between morphology and multiple traits of interest. Among these traits, the highest estimates of morphometricity (*m*^2^) were found for anthropomorphic traits, including birth weight (*m*^2^ = 0.19), current weight (*m*^2^ = 0.12) and height (*m*^2^ = 0.11). Significant *m*^2^ was also found for ten other traits of interest. However, it is important to note that the effects of these estimates were marginal, all below 5%, except for the area deprivation index (*m*^2^ = 0.06), and mothers' age at pregnancy (*m*^2^ = 0.05).

### 3.3 Model 2

The full outcome of Model 2 analyses are given I As evidenced by Fig. 4, estimates of $m^2$ and ${h^2}$ remained fairly stable whether using GREML or CORE GREML. Table 2 lists correlation estimates of the random effects and the outcome of likelihood ratio tests comparing Model 1 and Model 2. The likelihood-ratio tests indicated that the addition of a third component describing the covariance between the genomic and morphological effects did not result in a significant change in the goodness of fit for any of the traits. Significance tests of the correlation of random effects revealed no significant correlation of effects for any included outcome, even though some of the numerical estimates of the correlation were substantial (r > ± 0.5). This may indicate an issue concerning statistical power, and the results should be interpreted with some caution.

## 4 Discussion

Investigations of associations between observable traits and brain structure among rapidly maturing children and adolescents often yield inconsistent results. In the present paper, we adopted statistical methods from genetics (Zhou et al., 2020), and assessed the proportion of observable trait variance in children that can be explained by both similarity in common genetic variants and similarity in apparent cortical thickness. Most included traits showed moderate heritability. However, beyond anthropomorphic traits, our analyses revealed generally weak associations between cortical thickness and included traits. Further, an assessment of the covariance between genomic and morphological effects revealed no evidence of interdependence, suggesting that their contributions were unique.

### 4.1 Morphometricity

Our findings indicate the contribution of similarity in cortical morphology to the included traits were generally limited. This adds to recent literature suggesting that interindividual differences in cortical morphology share limited associations with behavioral differences among populations of normally developing children and adolescents (Genon et al., 2022). This may also extend to adults, where previous estimates of strong brain-behavior associations from small-scale studies have proved difficult to replicate in large-scale population-based samples (Botvinik-Nezer and Wager, 2022). Overall, our findings indicate that morphometricity is, as previously shown (Couvy-Duchesne et al., 2020, Fürtjes et al., 2023), reasonable for traits that are anthropometric in nature, such as height and weight, but this does not extend to measures of psychopathology or cognitive functions in this young population based sample. Couvy-Duchesne et al. (2020) specifically probed the association between anthropometrics and morphometricity and found that the morphometricity of traits from multiple different categories, such as symptoms of mental disorders, were in part attributable to body size. Birth weight, however, appeared in our sample to be unrelated to current height or weight. This indicates that birth weight may have associations with cortical morphology that are independent of later body size. Although we cannot determine the directionality of effects in the present study, previous studies indicate that low birth weight is associated with an enduring pattern of accelerated brain maturation (Karolis et al., 2017). In another recent study, Gilmore et al. (2020) showed that heterogeneity of cortical thickness at 6 years old is largely present at 1 year of age, highlighting the lasting importance of neonatal characteristics on later brain development, which continues into adulthood (Walhovd et al., 2023).

The correlation between neighborhood socioeconomic conditions (as measured by the ADI) and cortical thickness supports previous research demonstrating that socioeconomic conditions is recognized in the child brain (Hackman et al., 2021). This association appears to go beyond population stratification, which were included as fixed effects in our models. The cause of this association is not known, but recent papers based on material from the ABCD study suggest that the association between socioeconomic conditions and brain morphology is partly mediated by a lack of supportive psychosocial stimulation and a lack of healthy food options more frequently found in disadvantaged compared to more advantaged areas (Dennis et al., 2022, Tomasi and Volkow, 2021). We also observed that interindividual differences in cortical thickness was associated with maternal age at pregnancy. While our analysis does not inform us about the directionality of this effect, lower maternal age has previously been linked to disadvantaged socioeconomic conditions (Moore et al., 1993, Restrepo-Méndez et al., 2015). It is possible that this association is partly confounded by birth weight, which showed a significant association with cortical morphology in our sample, and has previously been linked to both socioeconomic markers and maternal age (Restrepo-Méndez et al., 2015). However, in the present sample, the correlations between birth weight and measures of neighborhood socioeconomic conditions were virtually non-existent, indicating that neighborhood socioeconomic conditions may have links to cortical morphology beyond gestational factors.

### 4.2 Heritability analyses

We found that the majority of traits included were moderately heritable, which can be used as a reference for future investigations of SNP-based heritability in the ABCD study. However, we would like to acknowledge that some estimates are at the lower end compared to what is commonly reported. This is particularly true for height, with our estimate being approximately one third of what is typically found in adults (Yengo et al., 2022). The comparatively lower estimates of heritability may possibly be attributed to the age of the sample, as the heritability of many traits tends to be lower during childhood before increasing throughout adolescence (Bergen et al., 2007). This is also the case for height, with heritability estimates increasing dramatically from 11–12 years onwards (Jelenkovic et al., 2016). Another possible issue explaining the somewhat lower estimates of SNP-based heritability is the racially and ethnically diverse nature of the ABCD sample. Significant heterogeneity in either the genotype or the trait across different race / ethnicity may cause a deflation of global SNP-heritability. This effect may be present even as principal components of genetic ancestry scores are added as covariates in LMMs (Li and Keating, 2014). We also would like to note that following QC the sample size of the present study is at the lower end of what is recommended for robust estimates of SNP-based heritability (Visscher et al., 2014), and possibly underpowered to detect significant heritability below 5%. Therefore, the estimates of heritability for included traits such as internalizing, externalizing, and sleep disturbance should be considered with some caution, as a bigger sample might be needed to adequately address SNP-based heritability in this range.

### 4.3 Covariance

We show that the covariance between the genomic and morphological effects on the trait of interest is not significantly different from zero, i.e. the effects appeared to be independent. This finding has two implications. Firstly, it indicates that our estimates of heritability and morphometricity may not be affected by the covariance between these factors. Secondly, it suggests that while cortical morphology is a heritable trait (van der Meer and Kaufmann, 2022), this does not necessarily translate into a pairwise similarity in cortical thickness between individuals who share genomic similarity, at least not conditioned on the traits included in the present study. Conceivably, the covariance of the effects of genomic and cortical thickness similarity might increase with age, as the influence of genetic factors on cortical morphology becomes stronger throughout adolescence (Schmitt et al., 2014). However, the lack of relationship between SNP-based and cortical thickness-based similarity, as evidenced by Fig. 3, could also indicate that the genetic units contributing to genetic similarity are not the same as the genetic units that contribute to similarity in cortical thickness (Boyle et al., 2017). Some care should be taken with this interpretation, however, due to the highly complex time- and location (i.e. region)-specific influence of genetic factors on cortical thickness (Kang et al., 2011, Strike et al., 2019, van der Meer and Kaufmann, 2022), which might not be captured well by a coarse similarity in cortical thickness across all vertices. The power to detect covariances in the present study might also be inadequate, as indicated by the substantial yet non-significant estimates of correlation found in Table 2. In the original CORE GREML paper by Zhou et al. (2020), ten traits with high heritability were selected to maximize the power to detect genome-transcriptome covariance. A recent paper by Owens et al. (2021) showed that small effect sizes are generally expected in the ABCD study, which might make sound inference regarding gene-morphology covariance complicated in this sample.

### 4.4 Limitations

The present paper has four limitations of particular importance. First, treating morphological and genetic effects as random avoids the issue of exhausting statistical power on hypothesis testing corrections for individual SNPs or vertices, but comes at the cost of spatial resolution, i.e. we cannot decipher which parts of the brain that contributed to variation in a trait.

Second, the present study is cross-sectional, representing only a snapshot of the child brain at a single point in time. It is possible that the link between individual similarity in cortical thickness and individual differences in traits is better understood looking at change over time (Foulkes and Blakemore, 2018, Rakesh et al., 2023), or that the sensitivity of cortical thickness to relevant outcome variables increases as individuals age (Mewton et al., 2022). A promising avenue might involve the calculation of separate BRMs for multiple timepoints and look for changes in interindividual differences in cortical thickness or other morphological measures across time, and how these changes relate to both changes in the influence of genetic factors and in observable traits.

Third, any type of neuroimaging measure can be expressed as a relatedness matrix. In the present paper, we limited our approach to cortical thickness. To better capture the strength afforded by the multimodal approach of large-scale imaging studies, future studies should seek to integrate the information afforded by multiple imaging derived phenotypes.

Fourth, the present study is to our knowledge the first to calculate estimates of morphometricity based on harmonized imaging data. Although generally recommended to account for site and scanner effects, it is conceivable that harmonization reduces individual differences in morphology, in turn reducing estimates of morphometricity. Couvy-Duchesne et al. (2020) also showed that smoothing cortical thickness may reduce estimates of morphometricity, and that no smoothing may be considered more powerful processing approach. However, in the present paper we did not compare estimates with and without smoothing. Lastly, we did not include any additional QC on the BRM, e.g., individuals with outlying values of brain similarity were left in the final sample. Future studies should seek to better assess how choices made during brain imaging processing, such as harmonization, smoothing and outlier removal, affect morphometricity estimates.

## 5 Concluding remarks

Here, we employed methods from statistical genetics to capture the association between cortical morphology and traits spanning the child phenome. Within the same linear mixed model framework, we assessed the effects of genetic similarity and its potential association with morphological similarity. Overall, associations with morphology were mostly limited to anthropometric traits, although some associations with neighborhood socioeconomic conditions were also observed. The estimated contribution of genetic effects to trait variance was at the lower end of what is commonly found, possibly attributable to the age and the racial / ethnic makeup of the sample. No significant covariance between the effects of cortical morphology and genetic effects was found. Future studies should seek to better integrate information from different imaging derived measures beyond cortical thickness.

## CRediT authorship contribution statement

**Alnæs Dag:** Supervision, Writing – review & editing. **Westlye Lars T.:** Funding acquisition, Supervision, Writing – review & editing. **Tamnes Christian K.:** Writing – review & editing. **Rodriguez-Cabello Sara F.:** Writing – review & editing. **Norbom Linn B.:** Data curation, Writing – review & editing. **Dahl Andreas:** Conceptualization, Data curation, Formal analysis, Visualization, Writing – original draft. **Eilertsen Espen M.:** Conceptualization, Writing – review & editing. **Lee Sang Hong:** Methodology, Software, Writing – review & editing. **Ystrom Eivind:** Conceptualization, Writing – review & editing. **Tandberg Anneli D.:** Writing – review & editing. **Leonardsen Esten:** Writing – review & editing.

## Declaration of Competing Interest

The authors declare that they have no known competing financial interests or personal relationships that could have appeared to influence the work reported in this paper.

## Data Availability

The authors do not have permission to share the data. However, an example of the analysis pipeline using simulated data is available at https://osf.io/uzp82/?view_only= 436a60d94542477b9ad0bb6868c0a7a9.
