# Supplementary material for: Genetic and brain similarity independently predict childhood anthropometrics and neighborhood socioeconomic conditions
Source: Dev Cogn Neurosci. 2024 Jan 4;65:101339. doi: 10.1016/j.dcn.2023.101339 (PMC10818201; doi:10.1016/j.dcn.2023.101339)
Supplement: Supplementary file 1 — Supplementary material [file mmc1.docx]

Supplementary Information for:

Authors: Andreas Dahl^a,b*^, Espen M. Eilertsen^c^, Sara F. Rodriguez-Cabello^a,b^, Linn B. Norbom^b,c^, Anneli D. Tandberg ^a,c^, Esten Leonardsen^a,b^, Sang Hong Lee^e,f^, Eivind Ystrom^c,g^, Christian K. Tamnes^b,c,d^, Dag Alnæs^b^, Lars T. Westlye^a,b,h^

^a^ Department of Psychology, University of Oslo, Oslo, Norway

^b^ NORMENT, Division of Mental Health and Addiction, Oslo University Hospital & Institute of Clinical Medicine, University of Oslo, Oslo, Norway

^c^ Research Center for Developmental Processes and Gradients in Mental Health (PROMENTA), Department of Psychology, University of Oslo, Oslo, Norway

^d^ Department of Psychiatric Research, Diakonhjemmet Hospital, Oslo, Norway

^e^ Australian Centre for Precision Health, UniSA Allied Health & Human Performance, University of South Australia, Adelaide, Australia

^f^ South Australian Health and Medical Research Institute (SAHMRI), University of South Australia, Adelaide, Australia

^g^ Department of Mental Disorders, Norwegian Institute of Public Health, Oslo, Norway

^h^ KG Jebsen Center for Neurodevelopmental Disorders, University of Oslo, Norway

Correspondence: AD ([andreas.dahl@psykologi.uio.no](mailto:andreas.dahl@psykologi.uio.no))

Scripts for the main analysis are available at https://osf.io/uzp82/?view_only=436a60d94542477b9ad0bb6868c0a7a9

**This file includes:**

Table S1 – S3

Figure S1 – S2

**Supplementary table S1:** Comparison of included and excluded participant on key demographic variables. Race / ethnicity groupings are based on caregiver report. Groupings were based on Barch et al. (2021) and defined based on whether the caregiver would describe the child as having a Hispanic ethnicity (ABCD element name *demo_ethn_v2*) and which racial identity(ies) that describe their child (“Yes” in ABCD element name *demo_race_a_p___10* to *25*). If a caregiver indicated that a child had Hispanic ethnicity, they were considered as Hispanic regardless of any indicated racial identity(ies). If a caregiver endorsed non-Hispanic ethnicity, children were grouped as Non-Hispanic White if only White racial identity was indicated (*demo_race_a_p___10*), as Non-Hispanic Black if only Black racial identity was indicated (*demo_race_a_p___11*), as Native American / Alaska Native if only Native or Native Alaska racial identity was indicated (*demo_race_a_p___12* & *demo_race_a_p___13*), as Asian if only Japanese, Chinese, Korean, Vietnamese, Filipino, Asian Indian or Other Asian racial identity was indicated (*demo_race_a_p___18* to *demo_race_a_p___24*), as Multi-racial if multiple racial identities were reported, or as belonging to the “Additional” grouping if Native Hawaiian, Guamanian, Samoan, Other Pacific or “Other” was indicated as racial identity (*demo_race_a_p___14* to *demo_race_a_p___17* and *demo_race_a_p___25*) , or if the caregiver refused to answer (*demo_race_a_p___77*).

|  |  |  | Excluded (N=4,116) |  | Included (N=7,760) |  | Eff.size difference |
| --- | --- | --- | --- | --- | --- | --- | --- |
| **Age** |  |  |  |  |  |  | -0.039ᵃ |
|  | Mean (SD) |  | 9.9 (0.6) |  | 9.9 (0.6) |  |  |
|  | Median (IQR) |  | 9.9 (1.2) |  | 9.9 (1.1) |  |  |
|  | Range |  | 8.9 - 11.1 |  | 8.9 - 11.0 |  |  |
| **Yearly household income** |  |  |  |  |  |  | 0.032ᵇ |
|  | $12,000 - $15,999 |  | 89 (2.16%) |  | 184 (2.37%) |  |  |
|  | $16,000 - $24,999 |  | 177 (4.30%) |  | 347 (4.47%) |  |  |
|  | $25,000 - $34,999 |  | 199 (4.83%) |  | 455 (5.86%) |  |  |
|  | $35,000 - $49,999 |  | 271 (6.58%) |  | 663 (8.54%) |  |  |
|  | $50,000 - $74,999 |  | 455 (11.05%) |  | 1044 (13.45%) |  |  |
|  | $75,000 - $99,999 |  | 457 (11.10%) |  | 1115 (14.37%) |  |  |
|  | $100,000 - $199,999 |  | 1000 (24.30%) |  | 2314 (29.82%) |  |  |
|  | $200,000 and greater |  | 422 (10.25%) |  | 828 (10.67%) |  |  |
|  | Missing |  | 1046 (25.41%) |  | 810 (10.44%) |  |  |
| **Race / Ethnicity** |  |  |  |  |  |  | 0.196 ᵇ |
|  | Non-Hispanic White |  | 1865 (45.31%) |  | 4343 (55.97%) |  |  |
|  | Hispanic |  | 857 (20.82%) |  | 1553 (20.01%) |  |  |
|  | Non-Hispanic Black |  | 686 (16.67%) |  | 1110 (14.30%) |  |  |
|  | Multi-racial |  | 322 (7.82%) |  | 485 (6.25%) |  |  |
|  | Native American / Alaska Native |  | 108 (2.62%) |  | 187 (2.41%) |  |  |
|  | Asian |  | 217 (5.27%) |  | 11 (0.14%) |  |  |
|  | Additional |  | 61 (1.48%) |  | 71 (0.91%) |  |  |
| **Sex** |  |  |  |  |  |  | 0.01ᵇ |
|  | Female |  | 1997 (48.52%) |  | 3683 (47.46%) |  |  |
|  | Male |  | 2119 (51.48%) |  | 4077 (52.54%) |  |  |
| ᵃ Cohen's d, unequal variances assumed (df=8131.6) | | | | | | | |
| ᵇ Cramer's V (df=1) | | | | | | | |

**Supplementary table S2:** Outcome of all Model 1 analyses.

|  | | **h²** | | | **m²** | | |
| --- | --- | --- | --- | --- | --- | --- | --- |
| **Trait** | **n** | **Estimate** | **SE** | **p** | **Estimate** | **SE** | **p** |
| Height | 7,163 | 0.190 | 0.044 | 0.000* | 0.110 | 0.013 | 0.000* |
| Weight | 7,745 | 0.134 | 0.044 | 0.005* | 0.123 | 0.013 | 0.000* |
| Birth weight | 7,439 | 0.064 | 0.042 | 0.157 | 0.189 | 0.016 | 0.000* |
| Age at pregnancy | 7,606 | 0.086 | 0.046 | 0.084 | 0.050 | 0.009 | 0.000* |
| Parent education | 7,657 | 0.166 | 0.051 | 0.002* | 0.032 | 0.008 | 0.000* |
| Area deprivation | 7,458 | 0.165 | 0.050 | 0.002* | 0.064 | 0.011 | 0.000* |
| Child opportunity index | 7,082 | 0.184 | 0.050 | 0.001* | 0.022 | 0.007 | 0.005* |
| Picture vocabulary | 7,620 | 0.200 | 0.048 | 0.000* | 0.014 | 0.005 | 0.011* |
| Flanker task | 7,580 | 0.075 | 0.048 | 0.147 | 0.007 | 0.005 | 0.177 |
| Working memory | 7,615 | 0.129 | 0.048 | 0.011* | 0.024 | 0.007 | 0.001* |
| Card sorting | 7,592 | 0.167 | 0.049 | 0.002* | 0.009 | 0.004 | 0.033* |
| Pattern recognition | 7,619 | 0.119 | 0.048 | 0.018* | 0.016 | 0.006 | 0.006* |
| Reading | 7,758 | 0.204 | 0.048 | 0.000* | 0.016 | 0.006 | 0.007* |
| Fluid intelligence | 7,656 | 0.189 | 0.048 | 0.000* | 0.033 | 0.007 | 0.000* |
| Crystallized intelligence | 7,626 | 0.241 | 0.048 | 0.000* | 0.021 | 0.006 | 0.001* |
| CBCL Internalizing | 7,584 | 0.040 | 0.047 | 0.451 | 0.005 | 0.004 | 0.204 |
| CBCL Externalizing | 7,732 | 0.019 | 0.047 | 0.691 | 0.014 | 0.005 | 0.012* |
| Pre-psychosis | 7,759 | 0.149 | 0.054 | 0.011* | 0.002 | 0.003 | 0.532 |
| Sleep disturbance | 6,750 | 0.018 | 0.046 | 0.691 | 0.001 | 0.002 | 0.770 |

**h²** = SNP-based heritability

**m²** = Morphometricity

***** Wald test (df=1) significant at p <.05 under the null hypothesis that the variance component is zero. False discovery rate adjusted.

**Supplementary table S3:** Outcome of all Model 2 analyses.

|  | | **h²** | | | **m²** | | |
| --- | --- | --- | --- | --- | --- | --- | --- |
| **Trait** | **n** | **Estimate** | **SE** | **p** | **Estimate** | **SE** | **p** |
| Height | 7,163 | 0.190 | 0.044 | 0.000* | 0.109 | 0.014 | 0.000* |
| Weight | 7,745 | 0.130 | 0.043 | 0.004* | 0.115 | 0.014 | 0.000* |
| Birth weight | 7,439 | 0.059 | 0.041 | 0.169 | 0.175 | 0.018 | 0.000* |
| Age at pregnancy | 7,606 | 0.076 | 0.043 | 0.105 | 0.041 | 0.009 | 0.000* |
| Parent education | 7,657 | 0.155 | 0.049 | 0.003* | 0.027 | 0.008 | 0.001* |
| Area deprivation | 7,458 | 0.156 | 0.048 | 0.003* | 0.059 | 0.011 | 0.000* |
| Child opportunity index | 7,082 | 0.180 | 0.050 | 0.001* | 0.021 | 0.007 | 0.009* |
| Picture vocabulary | 7,620 | 0.205 | 0.049 | 0.000* | 0.016 | 0.006 | 0.011* |
| Flanker task | 7,580 | 0.073 | 0.047 | 0.153 | 0.006 | 0.005 | 0.219 |
| Working memory | 7,615 | 0.130 | 0.048 | 0.011* | 0.025 | 0.007 | 0.001* |
| Card sorting | 7,592 | 0.159 | 0.047 | 0.002* | 0.007 | 0.004 | 0.095 |
| Pattern recognition | 7,619 | 0.121 | 0.048 | 0.018* | 0.017 | 0.006 | 0.009* |
| Reading | 7,758 | 0.207 | 0.049 | 0.000* | 0.017 | 0.006 | 0.009* |
| Fluid intelligence | 7,656 | 0.188 | 0.048 | 0.000* | 0.033 | 0.008 | 0.000* |
| Crystallized intelligence | 7,626 | 0.246 | 0.050 | 0.000* | 0.022 | 0.007 | 0.002* |
| CBCL Internalizing | 7,584 | 0.040 | 0.048 | 0.452 | 0.005 | 0.004 | 0.213 |
| CBCL Externalizing | 7,732 | 0.020 | 0.048 | 0.698 | 0.016 | 0.006 | 0.013* |
| Pre-psychosis | 7,759 | 0.149 | 0.055 | 0.011* | 0.002 | 0.003 | 0.543 |
| Sleep disturbance | 6,750 | 0.018 | 0.046 | 0.698 | 0.000 | 0.002 | 0.855 |

**h²** = SNP-based heritability

**m²** = Morphometricity

* Wald test (df=1) significant at p <.05 under the null hypothesis that the variance component is zero. False discovery rate adjusted.

**Supplementary figure S1**. Data processing flow.


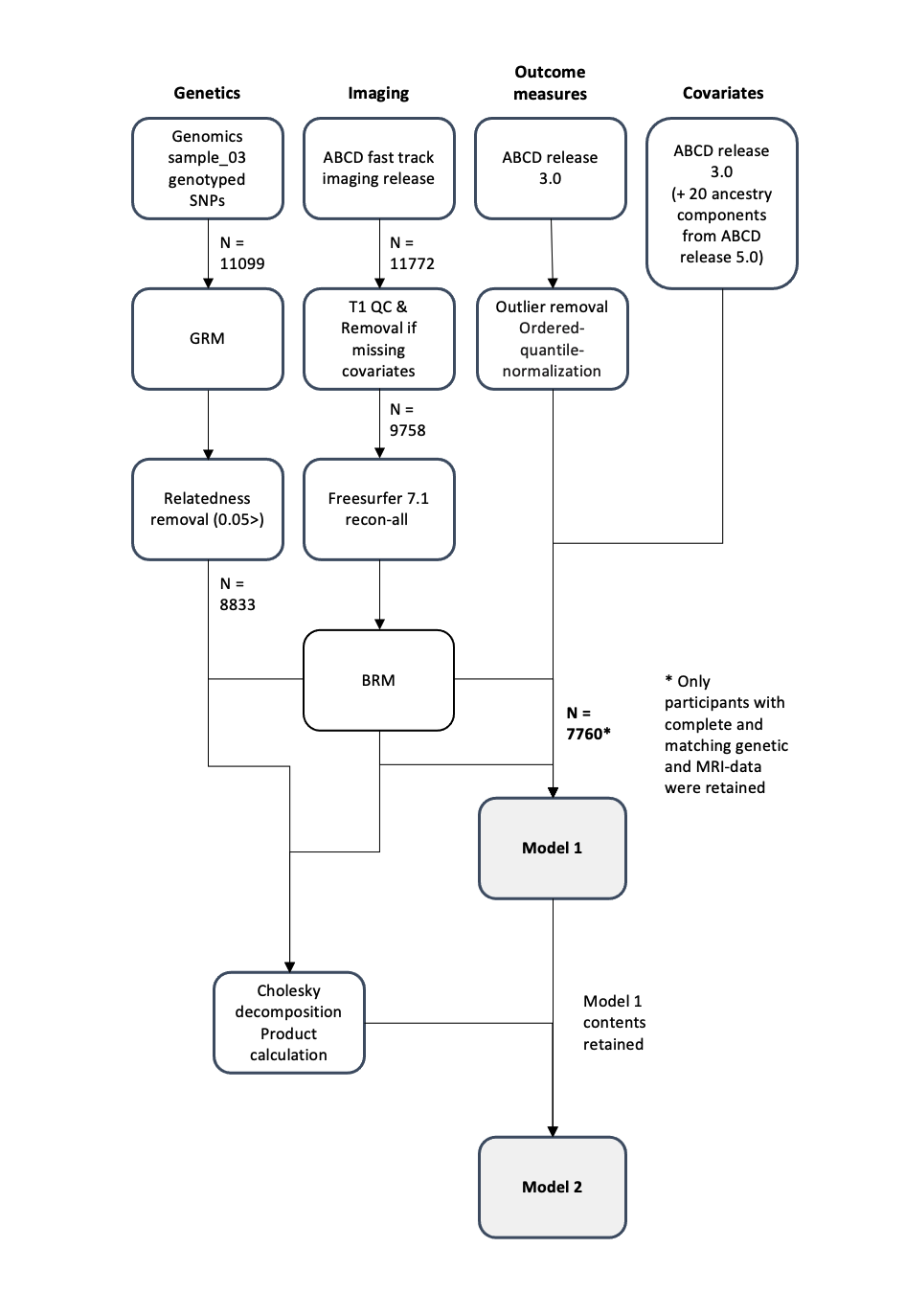


**Supplementary figure S2**. Mean cortical thickness for every participant at each scanner (colored) before (A) and after (B) the neuroCombat harmonization procedure.

*
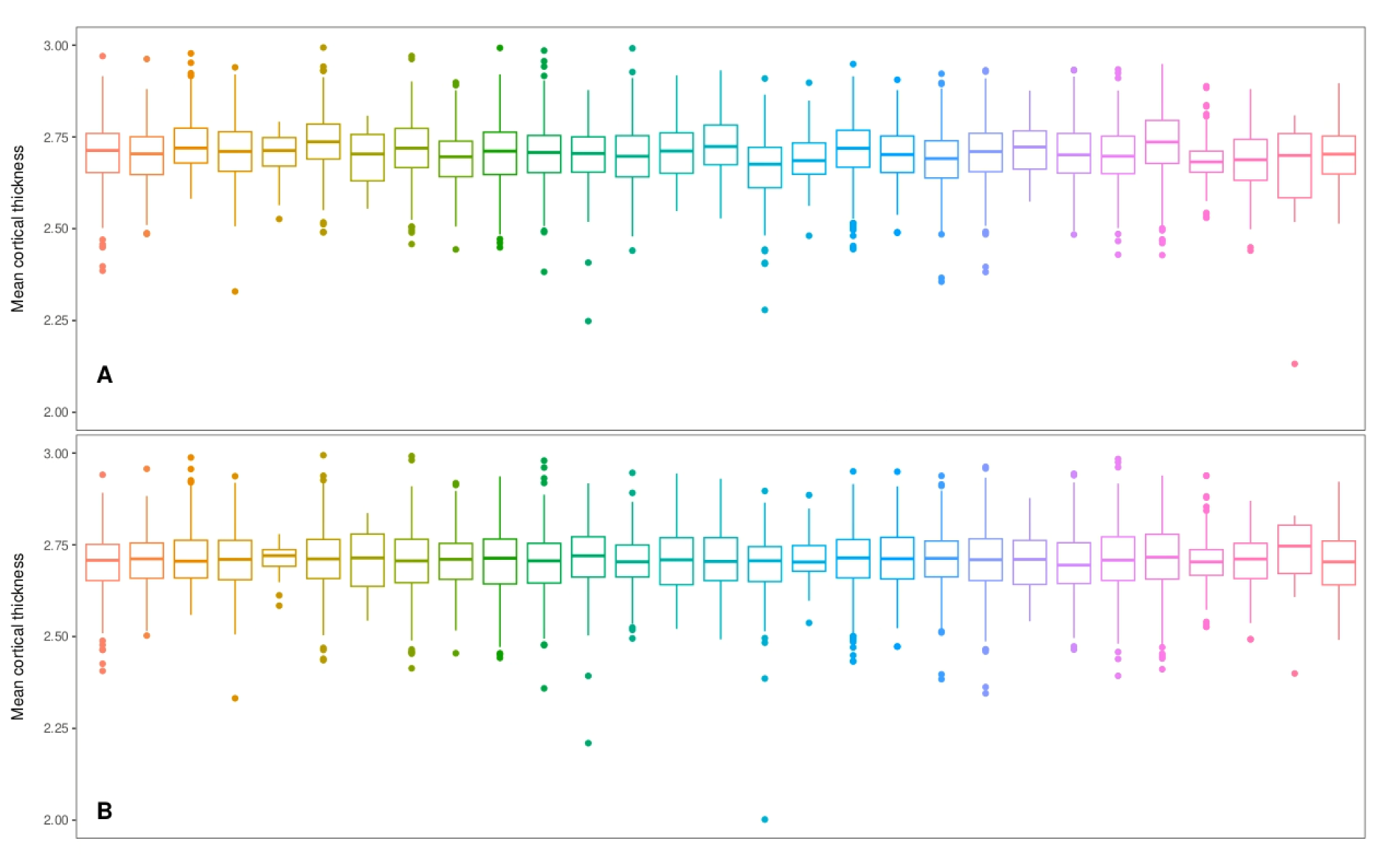
*

**References:**

Cohen, J. (1988). *Statistical power analysis for the behavioral sciences* (2nd ed). Hillsdale, N.J: L. Erlbaum Associates.

Barch, D. M., Albaugh, M. D., Baskin-Sommers, A., Bryant, B. E., Clark, D. B., Dick, A. S., ... & Xie, L. (2021). Demographic and mental health assessments in the adolescent brain and cognitive development study: Updates and age-related trajectories. Developmental cognitive neuroscience, 52, 101031
